# Optimized Solubilization of *Albizia myriophylla* Benth. Extract Using I‐Optimal Design for Anti‐*Streptococcus mutans* Activity

**DOI:** 10.1155/sci5/3315055

**Published:** 2026-07-23

**Authors:** Chaowalit Monton, Thaniya Wunnakup, Jirapornchai Suksaeree

**Affiliations:** ^1^ Drug and Herbal Product Research and Development Center, College of Pharmacy, Rangsit University, Lak Hok, Mueang Pathum Thani Pathum Thani, 12000, Thailand, rsu.ac.th; ^2^ Department of Pharmacognosy, College of Pharmacy, Rangsit University, Lak Hok, Mueang Pathum Thani Pathum Thani, 12000, Thailand, rsu.ac.th; ^3^ Medicinal Cannabis Research Institute, College of Pharmacy, Rangsit University, Lak Hok, Mueang Pathum Thani Pathum Thani, 12000, Thailand, rsu.ac.th; ^4^ Department of Pharmaceutical Chemistry, College of Pharmacy, Rangsit University, Lak Hok, Mueang Pathum Thani Pathum Thani, 12000, Thailand, rsu.ac.th

**Keywords:** *Albizia myriophylla*, dental caries, lupinifolin, mixture design, optimization, tooth decay

## Abstract

*Albizia myriophylla* Benth. is a medicinal plant possessing anti‐*Streptococcus mutans* activity, a key pathogen associated with dental caries, due to the presence of antimicrobial compounds such as lupinifolin. This study aimed to optimize a solvent system for solubilizing *A. myriophylla* extract. Initially, the anti‐*S. mutans* activity of the stem extract was determined, revealing minimum inhibitory and minimum bactericidal concentrations of 8 and 256 μg/mL, respectively. Five solvents—water, 95% ethanol, glycerin, polyethylene glycol (PEG) 400, and propylene glycol (PG)—were evaluated. The extract exhibited higher solubility in PG, followed by 95% ethanol and PEG 400. Based on these results, 95% ethanol, PG, and water were selected for mixture optimization using an I‐optimal design. A design space was constructed to ensure a minimum of 90% recovery of lupinifolin, achieving approximately 50% of the defined knowledge space. The optimal formulation for preparing a 5% *A. myriophylla* extract consisted of 95% ethanol, PG, and water in a mass ratio of 0.30:0.63:0.07. This formulation yielded a lupinifolin recovery ranging from 94.18% to 101.01%. A verification step confirmed the predictive accuracy and reliability of the model. Finally, the optimal formulation was evaluated for its anti‐*S. mutans* activity, which demonstrated effective antimicrobial properties. In conclusion, this study successfully identified a suitable solvent system for solubilizing *A. myriophylla* extract, which can serve as a guideline for the development of oral sprays targeting *S. mutans* in future research.

## 1. Introduction

The cumulative global prevalence of dental caries, a multifactorial disease driven primarily by microbial activity, poses significant public health challenges [1]. Among the myriad of pathogenic microorganisms involved, *Streptococcus mutans* stands out as a principal etiological agent due to its strong acidogenic and aciduric properties, leading to the demineralization of tooth enamel and subsequent carious lesions [2]. It operates within a complex oral biofilm environment, interacting with other organisms that together contribute to demineralization processes and the establishment of cariogenic microenvironments [3]. This highlights the need for effective antimicrobial strategies to maintain oral health and prevent caries caused by *S. mutans*.

Medicinal plants have long been recognized for their therapeutic properties, especially in traditional medicine systems where they are utilized for treating a range of ailments, including dental issues [4]. Plants offer a rich source of bioactive compounds with demonstrated antimicrobial activities that can potentially inhibit the growth of cariogenic bacteria. For instance, various studies have reported the antimicrobial efficacy of different herbal extracts against *S. mutans*, highlighting their potential as viable alternatives or adjunct therapies to conventional antimicrobials [5–8]. Among the medicinal plants with reported antimicrobial properties is *Albizia myriophylla* Benth., a member of the Fabaceae family. This plant has been utilized for its purported efficacy against a variety of conditions [9, 10]. Preliminary studies have showcased its potential against various pathogens, establishing a basis for exploring its applications within dental health, particularly against *S. mutans* due to the presence of lupinifolin in the plant [5, 9, 11, 12]. Previous studies have identified lupinifolin as a major antibacterial constituent of *A. myriophylla* with potent activity against *S*. *mutans*. Lupinifolin exhibited strong antibacterial efficacy, with low minimum inhibitory concentration (MIC) and minimum bactericidal concentration (MBC) values reported in earlier studies [9]. Variations in lupinifolin content among different sources of *A. myriophylla* wood were associated with differences in antibacterial potency, indicating that extracts containing higher amounts of lupinifolin demonstrated greater anti‐*S. mutans* activity [13]. Moreover, the antibacterial mechanism of lupinifolin has been reported to involve disruption of the bacterial cell wall and cytoplasmic membrane integrity, ultimately leading to bacterial cell death [14]. Due to its pharmacological relevance and association with antibacterial efficacy, lupinifolin may serve as a suitable phytochemical marker for quality control of *A. myriophylla* extracts and related formulations. These findings support the potential application of *A. myriophylla* in antimicrobial oral care products targeting cariogenic bacteria. However, the optimization of extract formulation and solubilization remains a crucial step that has not been extensively explored in the literature.

The choice of solvent for dissolving *A. myriophylla* extract is pivotal, as it directly influences the quality, efficacy, and bioactivity of the final extract. Solvent polarity plays a crucial role in determining the solubility of various phytochemical constituents, which can significantly affect their recovery and, subsequently, the extract’s therapeutic potential [15–17]. Different solvents can yield varying concentrations of bioactive compounds, with some demonstrating superior extraction capabilities for specific classes of phytochemicals [18, 19]. Thus, employing an optimal solvent system is essential not only for maximizing the yield of the desired phytochemicals but also for ensuring that the resulting extract retains its biological activity, thereby enhancing its potential application in therapeutic settings, including targeting *S. mutans* infection. Selecting the appropriate solvent ensures that the extract’s quality and efficacy remain intact, ultimately influencing its viability as a natural therapeutic agent. This is important not only for ensuring the appearance, quality, and efficacy of the extract and its final formulation, but also represents a key aspect to be further developed in future studies.

This study aimed to fill the identified gaps by exploring the optimized solubilization of *A. myriophylla* extract, with a focus on ensuring effective antimicrobial activity against *S. mutans*. The findings intend to contribute to the growing body of evidence supporting the use of herbal extracts in oral healthcare and to establish a framework for developing effective herbal therapies against bacterial pathogens associated with dental caries. By employing advanced statistical design techniques for extract solubilization optimization, the research seeks to align traditional knowledge with modern scientific methodologies.

## 2. Materials and Methods

### 2.1. Materials

Lupinifolin was obtained from the previous study [11]. 95% Ethanol, propylene glycol (PG), polyethylene glycol (PEG) 400, and glycerin were purchased from Krungthepchemi Co., Ltd., Bangkok, Thailand. All media used in antibacterial testing were purchased from HiMedia Laboratories LLC, PA, USA. Ultrapure water was produced from a Direct‐Q 3UV water purifier (Merck KGaA, Darmstadt, Germany). All AR grade and high‐performance liquid chromatography (HPLC) grade solvents (Fisher Chemical) were purchased from Apex Chemicals Co., Ltd., Bangkok, Thailand.

### 2.2. Extraction of *A. myriophylla* Stem

The dried stems of *A. myriophylla* were obtained from Charoensuk Osod, Nakhon Pathom, Thailand, on 6 March 2025. To verify the correct identification of the plant species and its specific part, an expert in plant taxonomy conducted an examination. The specimen was assigned the voucher code TMRC 074 and was deposited at the Drug and Herbal Product Research and Development Center, College of Pharmacy, Rangsit University. Subsequently, the plant material was ground into a coarse powder and stored in a dry environment until it was required for further use.

Ground *A. myriophylla* stem (2.4 kg) was macerated in 12 L of 95% ethanol for 3 days. The extract was collected, and the residual marc was subsequently macerated twice more using 8 L of 95% ethanol for 3 days each. All filtrates were combined and concentrated under reduced pressure at 45°C using a rotary evaporator to remove the solvent.

### 2.3. Determination of MIC and MBC of *A. myriophylla* Extract Against *S. mutans*

The MIC and MBC of *A. myriophylla* extract against *S. mutans* ATCC 25175 were evaluated to determine the suitable concentration of the *A. myriophylla* extract within a solvent system. *S. mutans* was first cultured overnight in brain heart infusion (BHI) broth under anaerobic conditions at 37°C. This culture was then diluted in BHI broth to meet the 0.5 McFarland standard and further diluted 100‐fold to achieve a concentration of 1 × 10^6^ CFU/mL. *A. myriophylla* extracts were prepared at a concentration of 200 mg/mL in methanol and then diluted in BHI broth to generate final concentrations ranging from 2 to 1024 μg/mL, ensuring that the maximum methanol concentration did not exceed 1%. A 100 μL aliquot of the *A. myriophylla* extracts was mixed with 100 μL of the bacterial suspension in each well of a sterile 96‐well plate (*n* = 3). The plates were incubated anaerobically at 37°C for 24 h. After incubation, 10 μL of 1 mg/mL resazurin was added to each well, and the plates were incubated for an additional 2 to 4 h at 37°C under aerobic conditions. The MIC was identified as the lowest concentration of *A. myriophylla* extracts at which the solution remained blue. Subsequently, 10 μL of these blue solutions was transferred onto BHI agar (*n* = 3) and incubated anaerobically at 37°C for 24 h. The MBC was defined as the lowest concentration of *A. myriophylla* extracts that showed no bacterial growth after incubation.

### 2.4. Determination of Inhibition Zone of *A. myriophylla* Extracts Against *S. mutans*

To confirm the appropriate concentration of the extract that dissolved in the solvent system, the disk diffusion method was used to evaluate the inhibition zone against *S. mutans*. Initially, *S. mutans* was cultured in BHI medium under anaerobic conditions at 37°C for 18–24 h. The bacterial density was then adjusted to the 0.5 McFarland standard using a 0.85% sodium chloride solution, and the inoculum was uniformly spread on BHI plates with a sterile swab. A volume of 40 μL of the *A. myriophylla* extract dissolved in methanol at various concentrations (0.1%, 0.25%, 0.5%, 1%, 5%, and 10% w/v) was applied to each 6‐mm sterile disk (*n* = 3) and allowed to dry. Additionally, methanol and a C20^®^ (0.12% chlorhexidine gluconate) product were also applied to separate disks (*n* = 3) and allowed to dry to serve as negative and positive controls, respectively. The disks were then placed on the agar plates and incubated under anaerobic conditions at 37°C for 24 h. The mean and standard deviation of the inhibition zones were recorded.

### 2.5. Selection of Solvent for the Solubilization of *A. myriophylla* Extract

The *A. myriophylla* extract was solubilized in a range of solvents to assess its suitability for future formulation applications. An excess amount of extract was dissolved in water, 95% ethanol, glycerin, PEG 400, or PG in 2‐mL microfuge tubes (*n* = 3). Each sample was subjected to ultrasonication for 10 min, followed by mixing with a vortex mixer (Vortex Genie 2, Scientific Industries, Inc., NY, USA) for 1 min, and then centrifuged at 6000 rpm for 5 min using an IKA mini G centrifuge (IKA Works (Thailand) Co. Ltd., Bangkok, Thailand). The supernatant from the center of each tube was collected, diluted to a concentration of 25 mg/10 mL with methanol, filtered, and subsequently analyzed for lupinifolin content using HPLC under the conditions previously reported [11]. The solubility of the extract, determined by measuring lupinifolin concentration, was calculated accordingly.

### 2.6. Optimization of Solvent System for the Solubilization of *A. myriophylla* Extract

The mass fractions of 95% ethanol, PG, and water were determined using an I‐optimal mixture design (Table 1) within Design‐Expert^®^ v. 11 (Stat‐Ease, Inc., MN, USA), applying the following constraints:

(1) 01.0≤A≤,

(2) 01.0≤B≤,

(3) 00.2≤C≤,

(4) A+B+C=1.0,

where A, B, and C represent 95% ethanol, PG, and water, respectively.

**TABLE 1:** Factors and responses of the I‐optimal mixture design for optimizing the solvent system used for *A. myriophylla* extract solubilization, expressed as mass ratios of 95% ethanol, PG, and water, and evaluated based on the percentage recovery of lupinifolin.

Std.	Run	Mass ratio (w/w)			Recovery of lupinifolin (%)
		95% ethanol	PG	Water	
2	1	0.77	0.23	0.00	102.83 ± 7.18
13	2	0.00	0.81	0.19	48.81 ± 2.84
14	3	0.45	0.35	0.20	95.48 ± 3.70
5	4	0.00	1.00	0.00	95.27 ± 11.81
7	5	0.91	0.00	0.09	83.81 ± 4.15
12	6	0.61	0.23	0.16	112.74 ± 18.29
15	7	0.16	0.64	0.20	72.89 ± 12.18
8	8	0.40	0.50	0.10	104.18 ± 5.01
1	9	0.27	0.73	0.00	106.00 ± 1.59
9	10	0.40	0.50	0.10	104.15 ± 8.79
3	11	0.77	0.23	0.00	109.70 ± 12.71
16	12	0.73	0.07	0.20	90.41 ± 1.22
11	13	0.40	0.50	0.10	87.33 ± 8.54
4	14	0.58	0.42	0.00	77.86 ± 3.98
6	15	0.91	0.00	0.09	69.70 ± 9.59
10	16	0.40	0.50	0.10	112.25 ± 4.38

*A. myriophylla* extract was solubilized according to the described methods, and subsequent HPLC analysis was conducted to quantify the lupinifolin content. The percentage recovery of lupinifolin was calculated relative to the concentration determined in 95% ethanol, which served as the extraction solvent for preparation of the crude extract and was therefore assigned a recovery value of 100%. Contour plots were subsequently generated to visualize the results. An analysis of variance (ANOVA) was conducted to assess the data. The optimization goal was set to achieve a recovery of lupinifolin of at least 90%. The optimal solvent system within the design space was then identified. To verify the predictive accuracy of the Design‐Expert^®^ model, *A. myriophylla* solutions were prepared in three separate runs. The model’s predictive accuracy was evaluated by calculating percentage errors, which compared the experimental results with the predicted values.

Furthermore, the inhibition zone of the optimal solvent system incorporating *A. myriophylla* extract was evaluated using the agar well diffusion method, following the previously described procedure. This assessment ensured the anti‐*S. mutans* activity of the optimal mixture, thus confirming its potential as a therapeutic option against *S. mutans*.

### 2.7. Statistical Analysis

The data were evaluated using ANOVA followed by a least significant difference (LSD) post hoc analysis to examine differences among multiple groups. This analysis was performed using IBM SPSS Statistics Version 22 (IBM Corporation, NY, USA). A *p* value of less than 0.05 was considered statistically significant at the 95% confidence level.

## 3. Results

Ethanolic extraction yielded a crude extract of 4.6% w/w, which appeared relatively low. The MIC and MBC values of *A. myriophylla* extract against *S. mutans* are shown in Table 2. The extract exhibited an MIC value of 8 μg/mL, while the MBC value was comparable to previously reported ranges. The MBC/MIC ratio exceeded 4–6. All antibacterial results were expressed as crude extract concentrations. Lupinifolin was used as the principal phytochemical marker for solubility evaluation throughout the present study.

**TABLE 2:** Minimum inhibitory concentration (MIC) and minimum bactericidal concentration (MBC) of *A. myriophylla* extract against *S. mutans*, expressed as crude extract concentrations in μg/mL.

Sample	MIC (μg/mL)	MBC (μg/mL)
*A. myriophylla* extract	8	256

**FIGURE 1:**
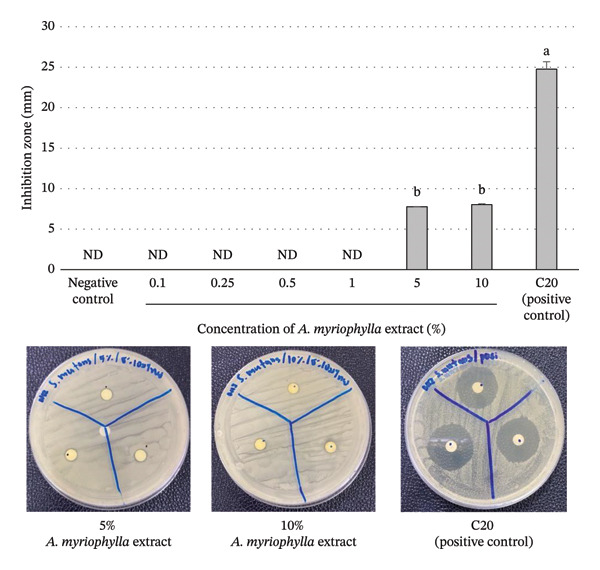
Inhibition zone of *A. myriophylla* extract at different concentrations against *S. mutans*, determined using the disk diffusion method. A volume of 40 μL of the extract dissolved in methanol at various concentrations (0.1%, 0.25%, 0.5%, 1%, 5%, and 10%) was applied to each sterile disk (*n* = 3) and allowed to dry before testing. Methanol and C20^®^ (0.12% chlorhexidine gluconate) were applied to separate disks and allowed to dry before use as negative and positive controls, respectively. Different letters above the bars indicate statistically significant differences (*p* < 0.05). ND = not detected. Representative images of the inhibition zones produced by the tested samples are shown in the lower panel as visual evidence of antibacterial activity.

The inhibition zones of *A. myriophylla* extract against *S. mutans* are presented in Figure 1, together with representative photographs of the disk diffusion assay. Extract concentrations of 5% and 10% produced comparable inhibition zones, leading to the selection of 5% for dissolution in the solvent system. This concentration corresponded to 6250‐fold MIC and 195‐fold MBC. In the present study, the 5% and 10% extract concentrations produced comparable inhibition zones without statistically significant differences, suggesting that increasing the extract concentration beyond 5% did not substantially enhance antibacterial activity under the tested conditions. Therefore, the 5% extract concentration was selected for subsequent solubility and formulation studies to minimize extract usage while maintaining detectable antibacterial activity.

**FIGURE 2:**
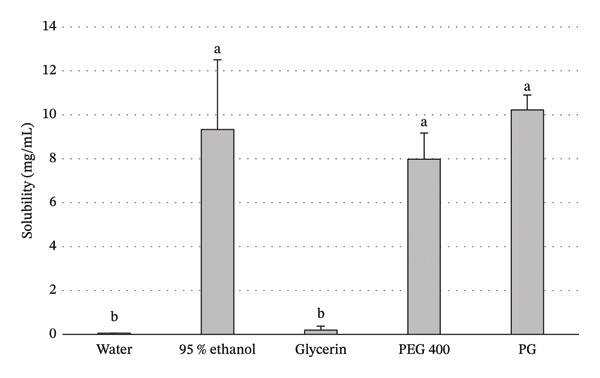
Solubility of *A. myriophylla* extract in various solvents, determined by HPLC analysis and expressed as lupinifolin concentration. Different letters above the bars indicate statistically significant differences (*p* < 0.05).

The solubility of *A. myriophylla* extract in different solvents, expressed as lupinifolin content, is shown in Figure 2. The calibration curve and representative HPLC chromatograms of lupinifolin are provided in the Supporting Information (Figures S1−S7). Water and glycerin demonstrated low solubility, whereas 95% ethanol, PEG 400, and PG showed higher solubilizing capacities. PG and 95% ethanol were selected for further optimization, with water also included for formulation purposes.

**FIGURE 3:**
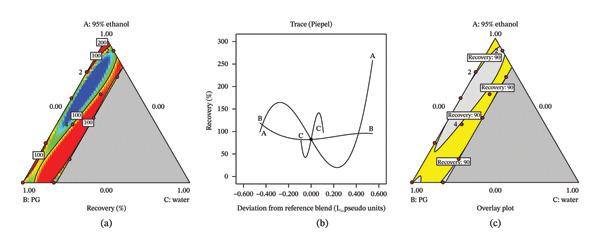
(a) Contour plots of percentage recovery of lupinifolin in various solvent systems composed of 95% ethanol, PG, and water. (b) Trace (Piepel) plot of solvent composition effects on lupinifolin recovery. (c) Design space for solvent compositions yielding ≥ 90% lupinifolin recovery.

Optimization using an I‐optimal mixture design restricted water content to a maximum of 0.2 mass fraction. The results of lupinifolin recovery are summarized in Table 1 and illustrated in Figure 3a, b. According to ANOVA (Table 3), the model was significant, with no significant lack of fit, supporting its predictive validity. The model demonstrated acceptable goodness of fit and predictive performance, with *R* ^2^, adjusted *R* ^2^, and predicted *R* ^2^ values of 0.9057, 0.7642, and 0.7554, respectively. In addition, the adequate precision value of 9.3459 indicated an adequate signal for navigating the design space.

**TABLE 3:** Analysis of variance (ANOVA) for the cubic model describing the percentage recovery of lupinifolin in the solvent system.

Source	Sum of squares	df	Mean square	*F* value	*p* value
Model	4351.7600	9	483.5300	6.4000	0.0174^∗^
Linear mixture	645.5800	2	322.7900	4.2700	0.0702
AB	600.1900	1	600.1900	7.9500	0.0304^∗^
AC	805.6500	1	805.6500	10.6700	0.0171^∗^
BC	784.8500	1	784.8500	10.3900	0.0181^∗^
ABC	776.2400	1	776.2400	10.2800	0.0185^∗^
AB (A‐B)	573.1400	1	573.1400	7.5900	0.0331^∗^
AC (A‐C)	828.5500	1	828.5500	10.9700	0.0162^∗^
BC (B‐C)	754.8200	1	754.8200	9.9900	0.0195^∗^
Residual	453.2000	6	75.5300		
Lack of fit	0.4073	1	0.4073	0.0045	0.9491
Pure error	452.7900	5	90.5600		
Cor total	4804.9600	15			

*Note:* A, B, and C represent 95% ethanol, propylene glycol (PG), and water, respectively. An asterisk (^∗^) denotes a statistically significant value.

The design space was defined to ensure at least 90% lupinifolin recovery (Figure 3c). The optimal solvent system was identified as 95% ethanol:PG:water in a mass ratio of 0.30:0.63:0.07. Validation with three independent lots confirmed that lupinifolin recovery approached 100% and closely matched predicted values with low percent error (Table 4). Recovery values slightly exceeding 100% were attributed to normal analytical and experimental variability associated with HPLC quantification and sample preparation.

**TABLE 4:** Verification of the predictive model by comparison between predicted and experimental values for percentage recovery of lupinifolin in the optimized solvent system.

Responses	Predicted values	Lot	Experimental values (*n* = 3)	Error (%)^∗^
Recovery of lupinifolin (%)	99.95	1	94.18 ± 3.62	−6.13
		2	98.79 ± 7.25	−1.17
		3	101.01 ± 1.05	1.05

^∗^Error (%) = (experimental value–predicted value) × 100/experimental value.

The *A. myriophylla* extract formulated in the optimal solvent system was re‐evaluated for anti‐*S. mutans* activity. The results demonstrated that the extract retained antibacterial properties, while the blank solvent system exhibited no inhibition (Table 5).

**TABLE 5:** Inhibition zones of the optimal solution containing 5% *A. myriophylla* extract, blank optimal solvent, and positive control against *S. mutans*, determined by the agar well diffusion method.

Sample	Diameter of the inhibition zone (mm, *n* = 3)
Optimal solution containing 5% *A. myriophylla* extract (40 μL)	11.70 ± 0.36
Blank optimal solvent (95% ethanol:PG:water at a mass ratio of 0.30:0.63:0.07, 40 μL)	Not detected
C20^®^ (0.12% chlorhexidine gluconate, 40 μL; positive control)	21.46 ± 0.55

## 4. Discussion

*A. myriophylla* extract is intended for incorporation into an oral spray formulation in future work. The present study focused on optimizing the solvent system to dissolve the ethanolic extract effectively. The relatively low extraction yield (4.6% w/w) compared with the previously reported microwave‐assisted extraction yield (6.7% w/w) from the previous study [11] may be attributed not only to differences in plant material but also to the extraction technique employed. The present study used conventional maceration, which generally provides lower extraction efficiency than microwave‐assisted extraction due to reduced cell wall disruption and mass transfer enhancement [20–22].

The MIC value of 8 μg/mL was four times lower than previously reported (32 μg/mL) [11], although the MBC remained similar. The relatively high MBC/MIC ratio may indicate a tendency toward bacteriostatic activity under the tested conditions [23]. However, because no time‐kill or kinetic studies were performed, definitive classification of the extract as bacteriostatic cannot be made, and this interpretation is based solely on the observed MBC/MIC ratio. In contrast, purified lupinifolin has previously been reported to exhibit bactericidal activity [11]. This difference suggests that antibacterial activity of the crude extract may also be influenced by other phytoconstituents. Although lupinifolin was selected as the principal phytochemical marker for solubility optimization and quality evaluation, other constituents may contribute to the overall antibacterial effect through additive or synergistic interactions. Comprehensive phytochemical characterization was beyond the scope of the present study, and further investigation is required to clarify the contribution of these constituents.

MIC and MBC are considered discrete quantitative variables because they are determined using serial two‐fold dilutions at fixed concentrations. In contrast, the disk diffusion method evaluates antibacterial activity based on radial diffusion through agar, which is influenced not only by antimicrobial potency but also by physicochemical properties such as compound diffusibility, solvent composition, and agar interaction [24, 25]. These factors may affect the size of the inhibition zone and limit direct quantitative comparison between concentrations.

Among the tested solvents, PG and 95% ethanol exhibited superior solubilization of lupinifolin, consistent with their known physicochemical properties. The inclusion of water, despite its poor solubility, was justified for safety, cost, and viscosity adjustment [26]. Notably, solvent effects on lupinifolin recovery differed from previous reports on cannabinoid solubilization [27], highlighting compound‐specific solubility behavior.

The optimized solvent system (95% ethanol:PG:water, 0.30:0.63:0.07) balanced solubility, safety, and practicality for potential oral spray development. Ethanol content was minimized to reduce mucosal irritation [28], while PG enhanced solubility, and water reduced viscosity to facilitate mass transfer [29, 30]. Validation confirmed that the model reliably predicted lupinifolin recovery, supporting its robustness for formulation design. The present study primarily focused on optimization of the solvent system based on lupinifolin recovery and confirmation of retained antibacterial activity after solubilization rather than direct pharmacological comparison between solvent systems. The extract dissolved in methanol was previously evaluated during preliminary antibacterial screening and concentration selection, whereas the optimized solvent system was subsequently assessed to confirm preservation of anti‐*S. mutans* activity after formulation. The high lupinifolin recovery together with the retained inhibition zone indicates that the antibacterial activity of the extract was maintained after solubilization. However, the optimization endpoint of the present study was lupinifolin recovery rather than direct antibacterial efficacy. Although antibacterial activity was retained after formulation, the relationship between lupinifolin recovery and overall antibacterial performance was not directly established. This represents a limitation of the present study and warrants further investigation using direct antibacterial efficacy as the optimization endpoint.

Importantly, the anti‐*S. mutans* activity of the optimized formulation was retained, confirming the antibacterial properties derived from the extract itself and not the solvent system. In the antibacterial assays, methanol and the blank optimized solvent system were used as negative controls to exclude solvent‐related effects, whereas C20^®^ containing 0.12% chlorhexidine gluconate was selected as a positive control due to its established antimicrobial use in oral care products. Nevertheless, a standard antibiotic comparator was not included because the present study focused primarily on formulation optimization and retention of antibacterial activity rather than direct pharmacological benchmarking against conventional antimicrobial agents. These findings support the potential applicability of incorporating *A. myriophylla* extract into oral spray formulations for further investigation and development. A limitation of the present study is that antibacterial activity was evaluated only against *S. mutans* under in vitro conditions. Biofilm‐based assays and simulation of the oral environment, including saliva interaction, pH variation, and retention within the oral cavity, were not investigated. Further studies evaluating antibiofilm activity, formulation stability, mucosal compatibility, and efficacy under simulated oral conditions are warranted to support future clinical applications.

## 5. Conclusions

This study successfully optimized a solvent system for solubilizing *A. myriophylla* extract, aiming to enhance the recovery of its active antimicrobial compound, lupinifolin, and support its application against *S. mutans*, a major causative agent of dental caries. The extract exhibited antibacterial activity, and solubility testing across several pharmaceutical solvents demonstrated that PG provided the greatest solubilizing capacity, followed by 95% ethanol and PEG 400. Based on these findings, a mixture optimization was conducted using a statistical design approach with 95% ethanol, PG, and water. This process established an optimal solvent ratio that provided high lupinifolin recovery, as confirmed by model verification. The optimized formulation maintained effective antibacterial activity against *S. mutans*, highlighting its potential for further pharmaceutical development. These findings offer a scientifically supported solvent system suitable for the efficient delivery of *A. myriophylla* phytoconstituents. The optimized formulation may serve as a basis for further development of oral care products, such as antibacterial sprays targeting *S. mutans*, pending additional studies under more clinically relevant conditions.

## Author Contributions

Chaowalit Monton: conceptualization, methodology, formal analysis, investigation, project administration, software, writing–original draft, writing–review and editing, resource, and supervision. Thaniya Wunnakup: methodology, formal analysis, investigation, and writing–original draft. Jirapornchai Suksaeree: methodology, formal analysis, investigation, and writing–original draft.

## Funding

The authors have nothing to report.

## Conflicts of Interest

The authors declare no conflicts of interest.

## Supporting Information

Additional supporting information can be found online in the Supporting Information section.

## Supporting information


**Supporting Information** Figure S1: Calibration curve of lupinifolin. Figure S2: HPLC chromatogram of lupinifolin (50 μg/mL). Figure S3: HPLC chromatogram of *A. myriophylla* extract in water. The water sample was analyzed without dilution, whereas the samples in Figures S4–S7 were diluted prior to HPLC analysis; therefore, peak intensities are not directly comparable. Figure S4: HPLC chromatogram of *A. myriophylla* extract in 95% ethanol. Figure S5: HPLC chromatogram of *A. myriophylla* extract in glycerin. Figure S6: HPLC chromatogram of *A. myriophylla* extract in PEG 400. Figure S7: HPLC chromatogram of *A. myriophylla* extract in PG.

## Data Availability

All data generated or analyzed during this study are included in this published article.
