# Supplementary material for: Optimized Solubilization of Albizia myriophylla Benth. Extract Using I‐Optimal Design for Anti‐Streptococcus mutans Activity
Source: Scientifica (Cairo). 2026 Jul 23;2026:3315055. doi: 10.1155/sci5/3315055 (PMC13392912; doi:10.1155/sci5/3315055)
Supplement: Supplementary file 1 — Supporting Information Figure S1: Calibration curve of lupinifolin. Figure S2: HPLC chromatogram of lupinifolin (50 μg/mL). Figure S3: HPLC chromatogram of A. myriophylla extract in water. The water sample was analyzed without dilution, whereas the samples in Figures S4–S7 were diluted prior to HPLC analysis; therefore, peak intensities are not directly comparable. Figure S4: HPLC chromatogram of A. myriophylla extract in 95% ethanol. Figure S5: HPLC chromatogram of A. myriophylla extract in glycerin. Figure S6: HPLC chromatogram of A. myriophylla extract in PEG 400. Figure S7: HPLC chromatogram of A. myriophylla extract in PG. [file SCI5-2026-3315055-s001.pdf]

Supplementary Material

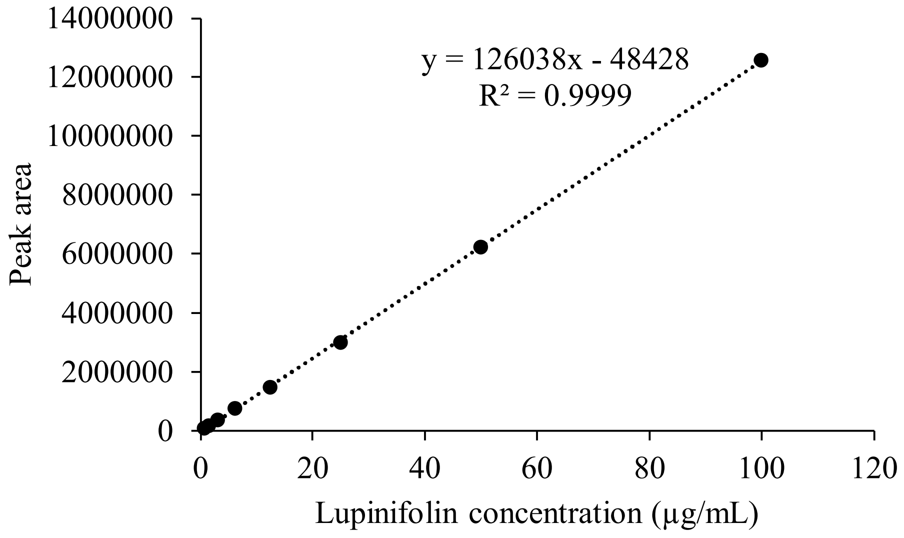

Fig. S1 Calibration curve of lupinifolin.

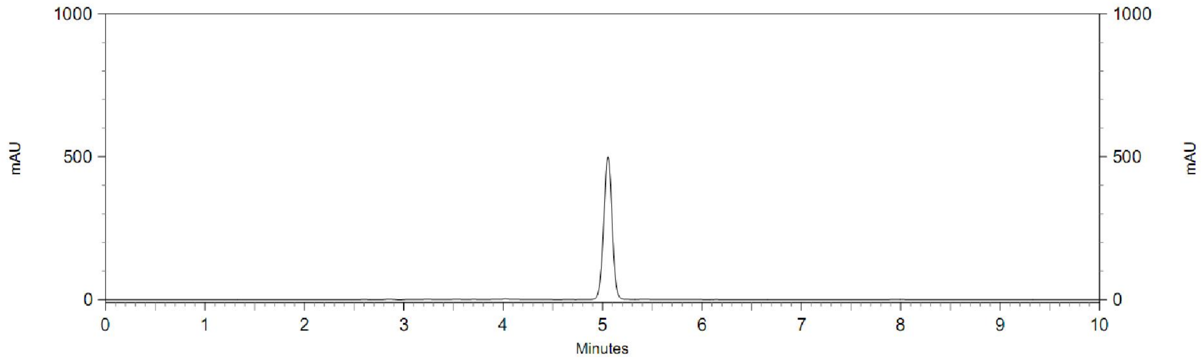

Fig. S2 HPLC chromatogram of lipinifolin (50 µg/mL).

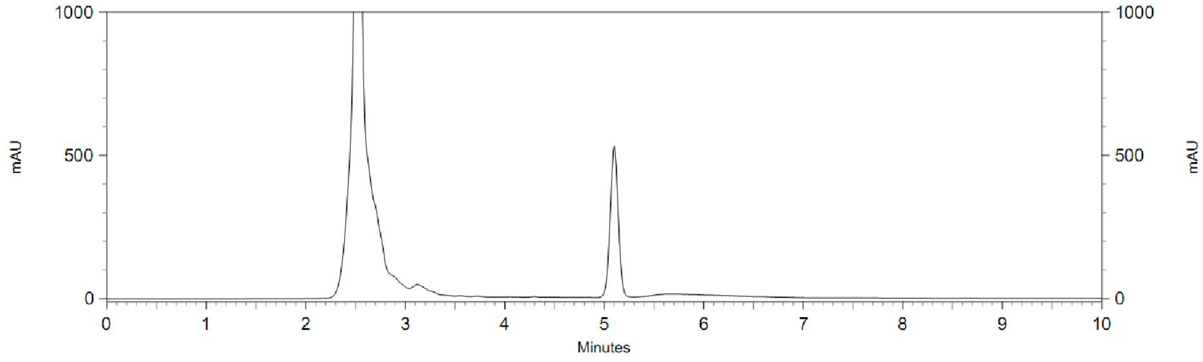

Fig. S3 HPLC chromatogram of *A. myriophylla* extract in water. The water sample was analyzed without dilution, whereas the samples in Figs. S4–S7 were diluted prior to HPLC analysis; therefore, peak intensities are not directly comparable.

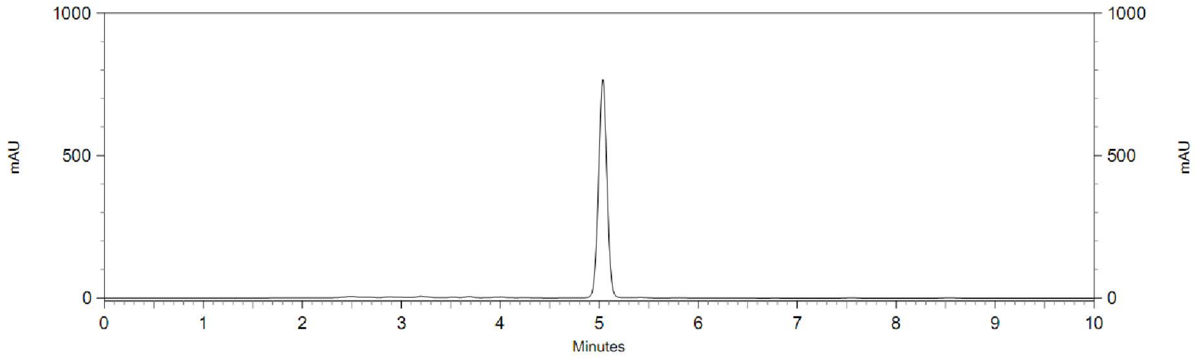

Fig. S4 HPLC chromatogram of *A. myriophylla* extract in 95% ethanol.

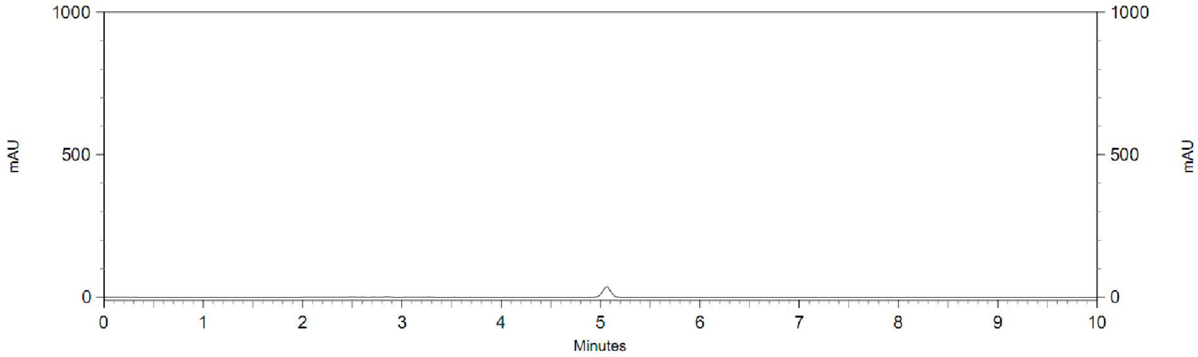

Fig. S5 HPLC chromatogram of *A. myriophylla* extract in glycerin.

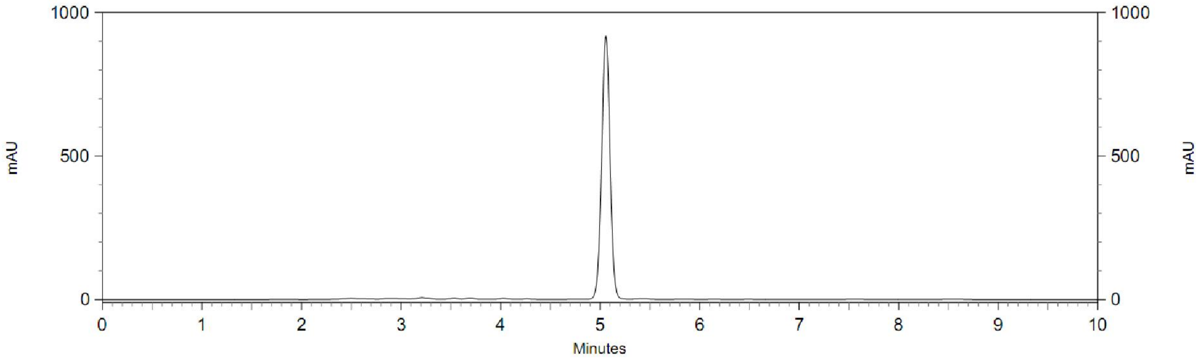

Fig. S6 HPLC chromatogram of *A. myriophylla* extract in PEG 400.

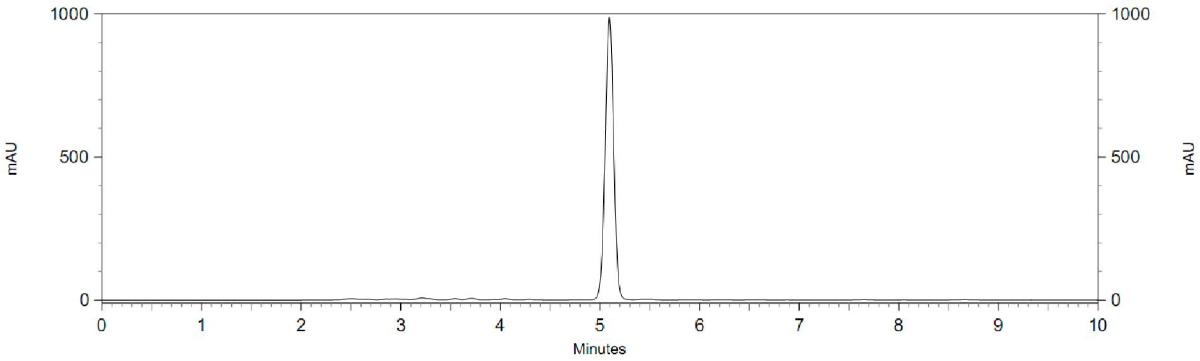

Fig. S7 HPLC chromatogram of *A. myriophylla* extract in PG.
